# Posttranslational modifications of titin from cardiac muscle: how, where, and what for?

**DOI:** 10.1111/febs.14854

**Published:** 2019-04-29

**Authors:** Franziska Koser, Christine Loescher, Wolfgang A. Linke

**Affiliations:** ^1^ Institute of Physiology II University of Muenster Germany

**Keywords:** cytoskeleton, elasticity, heart, heart disease, oxidation, oxidative stress, phosphorylation, protein kinase, protein phosphatase

## Abstract

Titin is a giant elastic protein expressed in the contractile units of striated muscle cells, including the sarcomeres of cardiomyocytes. The last decade has seen enormous progress in our understanding of how titin molecular elasticity is modulated in a dynamic manner to help cardiac sarcomeres adjust to the varying hemodynamic demands on the heart. Crucial events mediating the rapid modulation of cardiac titin stiffness are post‐translational modifications (PTMs) of titin. In this review, we first recollect what is known from earlier and recent work on the molecular mechanisms of titin extensibility and force generation. The main goal then is to provide a comprehensive overview of current insight into the relationship between titin PTMs and cardiomyocyte stiffness, notably the effect of oxidation and phosphorylation of titin spring segments on titin stiffness. A synopsis is given of which type of oxidative titin modification can cause which effect on titin stiffness. A large part of the review then covers the mechanically relevant phosphorylation sites in titin, their location along the elastic segment, and the protein kinases and phosphatases known to target these sites. We also include a detailed coverage of the complex changes in phosphorylation at specific titin residues, which have been reported in both animal models of heart disease and in human heart failure, and their correlation with titin‐based stiffness alterations. Knowledge of the relationship between titin PTMs and titin elasticity can be exploited in the search for therapeutic approaches aimed at softening the pathologically stiffened myocardium in heart failure patients.

## Graphical abstract

Titin is a giant elastic protein expressed in the contractile units of striated muscle cells, including cardiomyocytes. Rapid modulation of cardiac titin stiffness can be mediated through post‐translational modifications of titin, including oxidation and phosphorylation. This review notably details mechanically relevant phosphorylation sites in titin, their location, and the protein kinases and phosphatases known to target these sites. We include how titin phosphorylation correlates with titin‐based stiffness in heart failure.

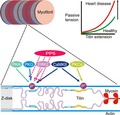

**Abbreviations**
AFM: atomic force microscopy
CaMKII: Ca^2+^/calmodulin‐dependent protein kinase II
cGMP: cyclic guanosine monophosphate
DCM: dilated cardiomyopathy
ERK: extracellular signal‐regulated kinase
FNIII: fibronectin type‐III
HCM: hypertrophic cardiomyopathy
HF: heart failure
HFpEF: heart failure with preserved ejection fraction
HT: hypertension
IDCM: idiopathic dilated cardiomyopathy
I/R: ischemia/reperfusion
Ig: immunoglobulin like
LA: left atrium (left atrial)
LV: left ventricle (left ventricular)
pI: isoelectric point
PAH: pulmonary arterial hypertension
PDE: phosphodiesterase
PKA: protein kinase A
PKG: protein kinase G
PP5: serine/threonine protein phosphatase 5
PPCM: peripartum cardiomyopathy
PTM: post‐translational modification
ROS/RNS: reactive oxygen/nitrogen species
RV: right ventricle (right ventricular)
SL: sarcomere length
TAC: transverse aortic constriction
TK: titin kinase
T2DM: type 2 diabetes mellitus
ZSF1: Zucker spontaneously hypertensive fatty‐1 (rat model)

## Introduction

**Figure 1:**
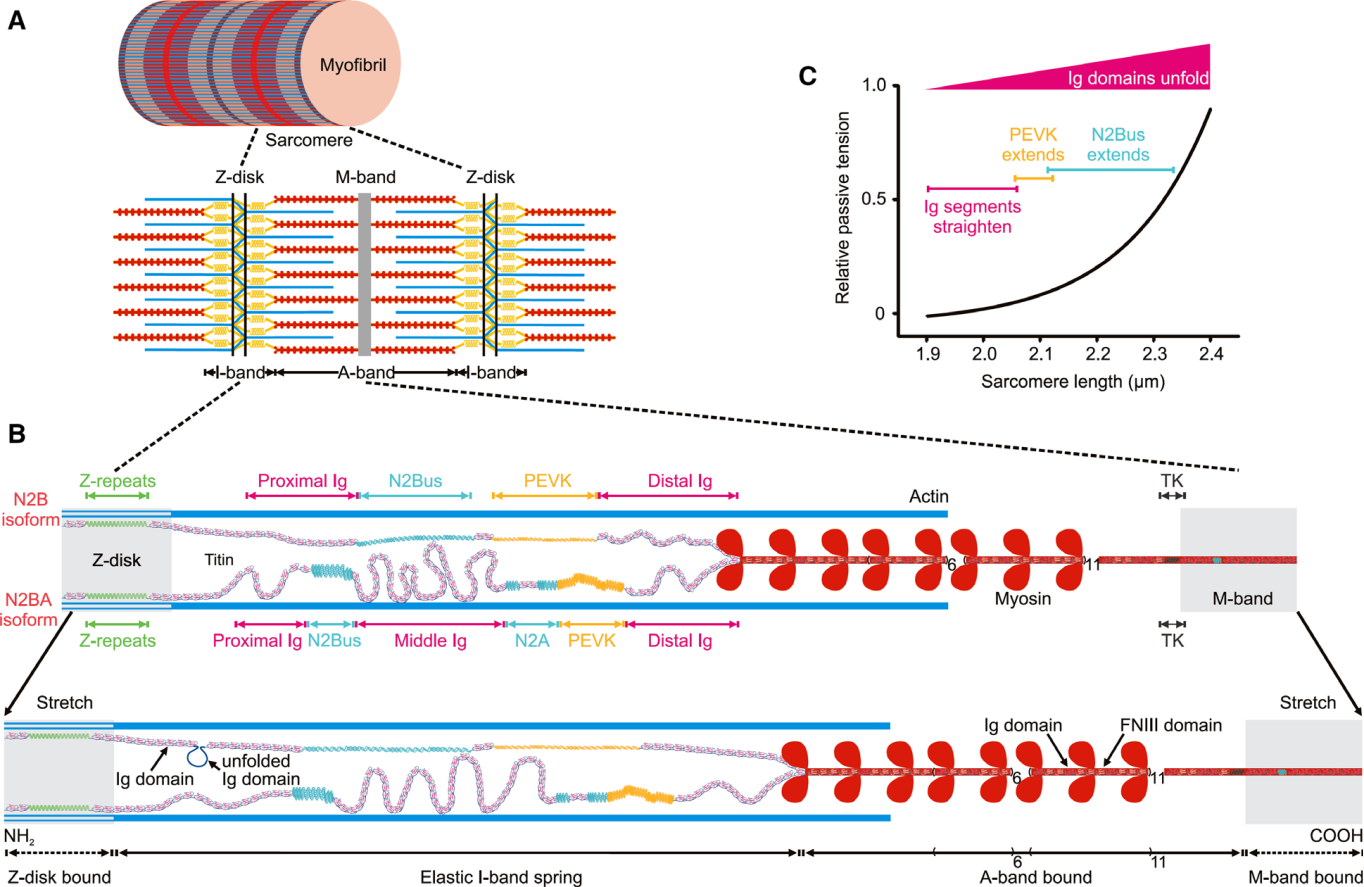
Titin isoforms and force‐extension mechanisms in human cardiac sarcomeres. (A) Schematic of a section of a myofibril constituted by sarcomeres (bordered by Z‐disks), which consist mainly of the three myofilaments, actin (thin), myosin (thick), and titin (elastic). (B) A half‐sarcomere is shown at two different stretch states. Cardiac titin isoforms, N2B and N2BA, are drawn as being coexpressed in the half‐sarcomere; molecular spring elements within elastic I‐band titin are highlighted. TK, titin kinase domain. (C) Relative titin‐based passive tension vs. sarcomere length relationship of a cardiomyocyte. Colors indicate the sequential extension of I‐band titin segments of the N2B isoform, which includes initial straightening of the Ig domain regions, followed by extension of the PEVK and N2Bus elements, and the continuous increase in the probability of Ig domain unfolding with stretching.

Throughout life, the heart is continuously adapting to varying mechanical demands imposed by the circulation. These demands are compensated for, at the level of the cardiomyocytes, by the contractile units known as the sarcomeres. Sarcomeres are serially arranged in myofibrils and (in a simplified view) consist of three main filament systems: actin‐based, myosin‐based, and titin‐based myofilaments (Fig. 1A). Actin and myosin generate force via the well‐known sliding‐filament mechanism, whereas the giant titin molecules (Fig. 1B) ensure sarcomeric integrity and provide myocytes with unique mechanical properties, as detailed below. Titin generates ‘passive’ tension through its spring‐like characteristics 1, 2, 3, which determine—along with those of the microtubular network 4, 5—the ‘passive’ stiffness of the cardiomyocyte and contribute a significant proportion of the total myocardial wall stiffness 6, 7. Moreover, titin stiffness and titin interactions with other myofilament proteins can modulate Ca^2+^‐dependent active tension 8. Therefore, alterations in titin‐based spring force affect both the passive and the active forces of cardiomyocytes. The modulation of titin spring force can occur via two principal mechanisms, the switch of titin isoforms and post‐translational modifications (PTMs), although additional mechanisms (e.g., binding of Ca^2+^ or chaperones) also play a role 9.

This review initially provides an updated model of titin extension in cardiac sarcomeres under stretch and highlights the importance of the unfolding of titin immunoglobulin‐like (Ig) domains in this process. The main goal of the review then is to explore how PTMs, specifically oxidation and phosphorylation, can alter titin‐based stiffness. Considering the expanding literature on titin phosphorylation, we include a comprehensive coverage of known, mechanically relevant, phosphosites within the titin spring segment and explain location‐specific effects of phosphorylation on titin stiffness mediated by different protein kinases and phosphatases. Our review also highlights gaps in knowledge that need to be filled. Since complex changes in titin phosphorylation have consistently been reported in heart disease, we discuss which of these changes are observed in which cardiac disorder, in both animal models of disease and human heart failure (HF), and how they alter cardiomyocyte passive force in failing vs. healthy hearts.

## Which springy regions in titin and molecular mechanisms of extension do we know?

In the sarcomere, titin molecules (Fig. 1B) extend from the Z‐disk (NH_2_‐termini) to the M‐band (COOH‐termini) 10. The titin protein is encoded by one of the largest genes, *TTN* (higher vertebrates have one titin gene). The *TTN* meta‐transcript encompasses 363 exons and 109224 base pairs (NCBI: http://www.ncbi.nlm.nih.gov/protein/NM_001267550.1) 11. Many exons are alternatively spliced resulting in multiple titin isoforms 11, 12, 13, with two main full‐length isoforms expressed in the heart (Fig. 1B): longer N2BA titin consisting of up to 313 exons and up to 34 350 amino acids (in human titin) and the shorter N2B variant, which includes exactly 191 exons and 26 926 amino acids (amino acid sequences of human titin according to UniProtKB entry http://www.uniprot.org/uniprot/Q8WZ42). Titin molecular segments are organized pursuant to the sarcomere structure and are referred to as Z‐disk‐bound element, elastic I‐band spring, A‐band‐bound element, and M‐band‐bound element (Fig. 1B). These segments consist of Ig domains interspersed with ‘Z’‐repeats (Z‐disk segment) or with other short unique sequences (Z‐disk/I‐band/M‐band segments), Ig domains alternating with fibronectin type‐III domains in ‘super repeat’ patterns (A‐band segment), a titin kinase (TK) domain (near the M‐band), and long stretches of Ig domains flanking long unique sequences, such as PEVK (made up of protein motifs rich in proline, glutamate, valine, and lysine) and N2Bus (I‐band) 12. N2Bus is found in cardiac but not in skeletal muscle titin isoforms. The PEVK segment but not N2Bus is exposed to massive alternative splicing. These unique sequences are considered to be intrinsically disordered and are one of two types of extensible segments in the elastic I‐band spring. The second type is tandem Ig domain regions termed ‘proximal Ig’, ‘middle Ig’, and ‘distal Ig’. The proximal and distal Ig segments are constitutively expressed, whereas the middle Ig region is alternatively spliced in N2BA as compared to N2B titin. As a result, there is a greater number of Ig domains and PEVK motifs in N2BA than in N2B isoforms (Fig. 1B). All of these extensible elements in cardiac I‐band titin contribute to the spring force generated by the elastic segment upon sarcomere stretch 14, 15.

The elasticity of titin's I‐band segment is entropic in nature 16, 17. The I‐band spring elements behave like serially linked biopolymers, generating entropic force upon stretching due to their tendency to maintain a more compact state rather than extended states. According to models of entropic biopolymer elasticity 16, 17, 18, 19, I‐band titin will resist against external stretch forces by generating an elastic force, which rises with the stretch amplitude and thus, the sarcomere length (SL), in a quasi‐exponential manner (Fig. 1C). Notably, at shorter SLs and low stretch forces (up to ~ 1 pN/titin molecule), titin becomes extended first due to the straightening of the short linkers between the tandem Ig domains 14, 15 (Fig. 1B,C). Intermediate to high stretch forces (up to ~ 10 pN/titin) thereafter extend the PEVK segment, followed by the N2Bus element 14, 15, 18, 19, 20, 21 (Fig. 1C). The wormlike chain model of entropic elasticity readily describes this sequential extension mechanism of cardiac titin 15, 18, 19.

In addition to I‐band segment extension, reversible Ig domain unfolding 22 contributes to titin extension under physiological stretch forces. Earlier, the unfolding of Ig domains was proposed as a safety mechanism, limiting high forces in the sarcomere but not taking place at physiological SLs and stretch forces 17, 18, 23. However, recent findings have confirmed, at both the molecular and sarcomere levels, that unfolding of Ig domains already occurs at lower, physiological forces below ~ 10 pN/titin 24, 25 (Fig. 1B,C). Interestingly, Ig unfolding provides a mechanism to store elastic energy, which is released by refolding 26, potentially assisting active muscle contraction 24 (titin's role in active muscle contraction is discussed in detail in reference 8). In conclusion, reversible Ig domain unfolding is a physiologically relevant mechanism of titin extension‐release and sarcomeric force adjustment.

## How can cardiac titin stiffness be modulated dynamically?

Since titin stiffness is a main contributor to cardiomyocyte stiffness and thus, total myocardial stiffness 27, 28, the modulation of titin stiffness will affect the wall tension of the heart chambers, with consequences for diastolic filling and subsequent systolic pump function (via an autoregulatory mechanism known as the Frank–Starling law) 8, 29, 30. One important long‐term mechanism of titin stiffness adjustment is titin isoform switching. It is based on the lower stiffness of the N2BA isoform as compared to the N2B isoform and the coexpression of these two isoforms in the same (half‐)sarcomere (Fig. 1B) 13, 31, 32. For instance, predominant expression of shorter, stiffer N2B titin is seen in adult as opposed to fetal hearts, which express long and compliant (fetal) N2BA isoforms; therefore, adult cardiac sarcomeres are much stiffer than fetal ones 33, 34, 35. The titin isoform composition is under the control of splicing factors, of which RNA‐binding motif 20 (RBM20) is the best‐known splicing factor for cardiac titin 36. The splicing repressor activity of RBM20 is likely to be regulated by different mechanisms; for example, it is inhibited by polypyrimidine tract‐binding protein 4 37. Additionally, thyroid hormone T3 and insulin have been shown to modulate myocardial titin isoform composition via the PI3K‐AKT‐mTOR kinase axis 38, 39. Their mechanism of action includes effects on RBM20 40, 41. Titin isoform switching allows more stable, general changes in titin‐based passive stiffness over a relatively long period. Isoform transitions of titin can also occur under pathological conditions, such as in endstage failing human hearts 42, 43, 44.

However, to compensate beat‐to‐beat requirements, the heart also needs quicker, finer control over passive tension. This can be achieved through PTMs of titin spring elements. Multiple lines of evidence have established that titin‐based passive stiffness can be readily adjusted via PTMs targeting the N2Bus and PEVK unique sequences, and perhaps also the Ig domains. In the following sections, this regulation via PTMs will be discussed, with consideration given to oxidation and emphasis put on phosphorylation.

## How do redox modifications affect titin‐based cardiomyocyte stiffness?

**Figure 2:**
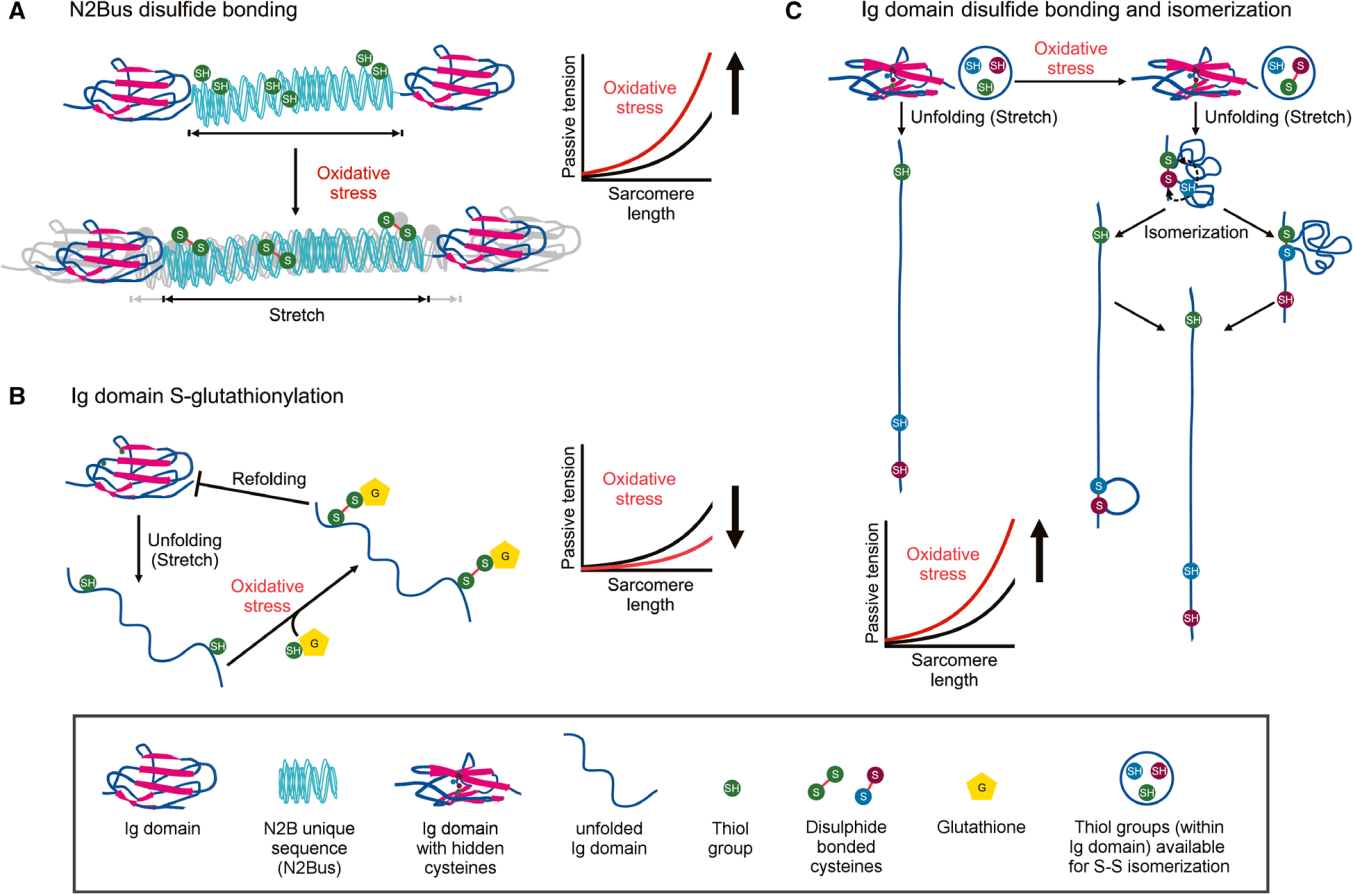
Mechanisms of titin‐based passive tension modulation by oxidative stress‐induced titin modifications. (A) Formation of up to three intramolecular disulphide bonds within the human N2Bus element of titin under oxidative stress increases titin‐based passive tension in cardiomyocytes. (B) Ig domain unfolding due to sarcomere stretching causes exposure of hidden (‘cryptic’) cysteines in Ig domains, which can become S‐glutathionylated under oxidative conditions. S‐glutathionylation prevents Ig domain refolding, resulting in decreased titin‐based passive tension. (C) Isomerization of disulfide bonds of the cysteine triad in titin Ig domains can occur under oxidative conditions. Depending on where the intramolecular S‐S cross‐linking occurs, titin‐based passive tension increases by different amplitudes. The key on bottom explains the different shapes and colors shown in the figure.

All three filament systems of the sarcomere are targeted by oxidative modifications (see 45 for details on actin and myosin modifications). Oxidative stress occurs when there is a disturbance in the balance between the production of reactive oxygen/nitrogen species (ROS/RNS) and antioxidant defense mechanisms 46. Depending on their nature and level, ROS/RNS can cause reversible and/or irreversible protein modifications mainly targeting amino acids containing a thiol group (predominantly cysteines but also methionine) 47, 48, 49, 50. Reversible oxidative modifications have been described for titin, including disulfide bonding in the N2Bus segment, S‐glutathionylation in Ig domains, and disulfide bonding and isomerization in Ig domains (Fig. 2). These modifications have been shown to alter titin‐based stiffness *in vitro* and in isolated cardiac muscle preparations 45.

### N2Bus oxidation

Indirect evidence for the possibility of oxidative modifications in the N2Bus titin segment was already obtained two decades ago. By immunoelectron microscopy, the extensibility of the N2Bus segment in cardiac sarcomeres appeared to be impaired under oxidizing conditions 15, 21. This finding was confirmed and extended later in single‐molecule force‐extension measurements using the atomic force microscope (AFM), which directly showed increased extensibility of recombinant N2Bus protein under reducing vs. oxidizing conditions 51 Moreover, this study demonstrated that up to three disulfide bridges could be formed by the six cysteines present in the human N2Bus segment (Fig. 2A). The disulfide bridges mechanically stabilize (cross‐link) the otherwise disordered N2Bus segment, which results in increased passive tension under oxidative stress conditions (Fig. 2A) 51, 52.

Disulfide bridge formation in the cardiac N2Bus segment might also interfere with intracellular signaling pathways intersecting with N2Bus 53. For example, disulfide bridges could alter the N2Bus‐binding propensity of the four‐and‐a‐half LIM‐domain proteins FHL1 and FHL2 54, 55 and the small heat shock proteins αβ‐crystallin and HSP27 56, 57, as well as the ATP‐dependent chaperone HSP90 58. One or more of these alterations could then affect mechanisms of mechanosensation and protein quality control in the cardiomyocytes under oxidative stress conditions, in addition to altering titin‐based passive tension. Another likely scenario is that redox modifications of N2Bus affect other types of PTMs in N2Bus, such as phosphorylation, with consequences for titin‐based passive tension modulation (see below). Preliminary evidence for such an interdependence has been published 59.

### Ig domain oxidation

The Ig domains of titin have been known for some time to contain putative sites of oxidation 60. Recent work has demonstrated that many titin Ig domains can be modified under oxidizing conditions by S‐glutathionylation and disulfide bonding/isomerization. Various studies used single‐molecule mechanical methods, such as AFM force spectroscopy, to elucidate the relationship between domain oxidation and mechanical strength of recombinantly expressed domains 61, 62, 63, 64, 65. In combination with human cardiomyocyte mechanics, it was shown that upon stretching cardiac sarcomeres and unfolding titin Ig domains, these domains reveal ‘cryptic’ cysteines, which are still buried inside the folded domain 61. The now‐exposed cysteines can become S‐glutathionylated under oxidative conditions, which prevent domain refolding and decrease the mechanical stability of the Ig domain (Fig. 2B). By inhibiting Ig domain refolding, this S‐glutathionylation causes the titin spring to become longer, thereby contributing to a decrease in cardiomyocyte passive stiffness (Fig. 2B), which, however, is readily reversible 61. S‐glutathionylation of sarcomere proteins, including titin, increases in murine hearts upon myocardial infarction 66, suggesting that S‐glutathionylation is a relevant mechanism of cardiomyocyte stiffness regulation under oxidative stress.

Recently, S‐sulfenylation of cryptic cysteines in Ig domains was demonstrated as a potentially ‘competing’ mechanism, as it can cause titin stiffening 62. Sulfenic acid is a short‐lived intermediate that either triggers protein misfolding or leads to the formation of a disulfide bond which protects the Ig domain fold. The covalent bond of the disulfide bridge cannot be broken by physiological levels of force/molecule (in the piconewton range). Therefore, the presence of an intramolecular S‐S bond leads to less stretchable (stiffer) Ig domain segments, as shown in a series of elegant experiments using single‐molecule force spectroscopy methods 64, 65. Such a mechanism would be expected to increase titin‐based passive stiffness in a reversible manner depending on the redox state of the cardiomyocyte. Currently there is no evidence to suggest whether S‐sulfenylation is a mechanism of titin stiffness regulation *in vivo*.

A related mechanism of stiffness modulation under oxidative stress is the formation and isomerization of disulfide bonds in unfolded titin Ig domains (Fig. 2C). Multiple alignments of Ig domain sequences of human titin (UniProKB entry http://www.uniprot.org/uniprot/Q8WZ42-1; isoform1; April 18, 2012; Version 4) revealed that 21% of the Ig domains present in elastic I‐band titin contain a conserved cysteine triad, which has the potential to form S‐S bridges and isomerize 63. Those Ig domains containing the conserved cysteine triad can be oxidized resulting in a disulfide bridge, which then becomes isomerized under force in a two‐step mechanism (Fig. 2C). This differs from the situation in reduced Ig domains, in which stretch causes all‐or‐none extension. Since disulfide bond formation and isomerization prevent further unfolding of the Ig domain, titin‐based passive tension is predicted to be increased (Fig. 2C) 63. Mechanical measurements on recombinant Ig domains of the middle Ig region by AFM force spectroscopy confirmed such a scenario 63. The S‐S isomerization provides an additional, potential mode of titin stiffness adjustment, because the ‘free’ length of the unfolded Ig domain is shorter or longer, depending on the position of the intramolecular S‐S cross‐link formed.

These mechanisms of oxidative modification of titin Ig domains depend on the accessibility of the cryptic cysteines buried inside the folded Ig domains. Since reversible Ig domain unfolding occurs at physiological forces 24 (Fig. 1C), cryptic cysteines should become accessible at least at higher physiological SLs. Notably, Ig domains containing the conserved cysteine triad are located almost exclusively in the middle Ig region, which is expressed in cardiac titin isoform N2BA (and skeletal isoform N2A) but not in N2B. Due to this inclusion of many more Ig domains (and PEVK motifs) in N2BA compared to N2B titin, the stretch force experienced by the N2BA spring (coexpressed with N2B in the same sarcomere; Fig. 1B) is much less than that experienced by the N2B spring. It is, therefore, likely that only very few (if any) Ig domains from the middle Ig segment unfold under physiological stretch forces and expose cryptic cysteines. Taken together, the mechanically relevant types of oxidative modification of titin Ig domains in the presence of physiological stretch forces and oxidative stress may be S‐glutathionylation and disulfide bonding (in unfolded Ig domains from the N2B isoform), rather than S‐S isomerization. Interestingly, S‐glutathionylation and disulfide bonding in titin Ig domains have opposite effects on overall titin stiffness, which implicates a unique potential for titin oxidation to differentially fine‐tune cardiomyocyte tension under oxidative stress. However, at present there is no information on the preferred type of titin oxidative modification and their effect on passive tension in the living myocardium.

In the muscle mechanics field, the consequences of titin oxidation and disulfide bond formation for the mechanical properties of the myocytes have only rarely been considered. For instance, many studies utilize antioxidants and reducing agents during tissue preparation and this type of pretreatment has the potential to break disulfide bonds and alter titin‐based stiffness prior to any experimental procedures or measurements. Therefore, the role oxidation plays in dynamic stiffness regulation of titin *in vivo* may be greater than currently understood.

## Which protein kinases/phosphatases (de)phosphorylate titin for a mechanical effect?

Phosphorylation is one of the most common PTMs that regulates cellular function. A variety of physiological stimuli, including hormones and neurotransmitters, regulate the cardiac contractile performance via phosphorylation/dephosphorylation of intracellular proteins, and sarcomere proteins are no exception 67, 68. A fine‐tuned system of protein kinases and protein phosphatases ensures the regulation of sarcomere function by phosphorylation. Some of these enzymes exert their specific effects through defined subcellular localization 58, 69, 70.

**Figure 3:**
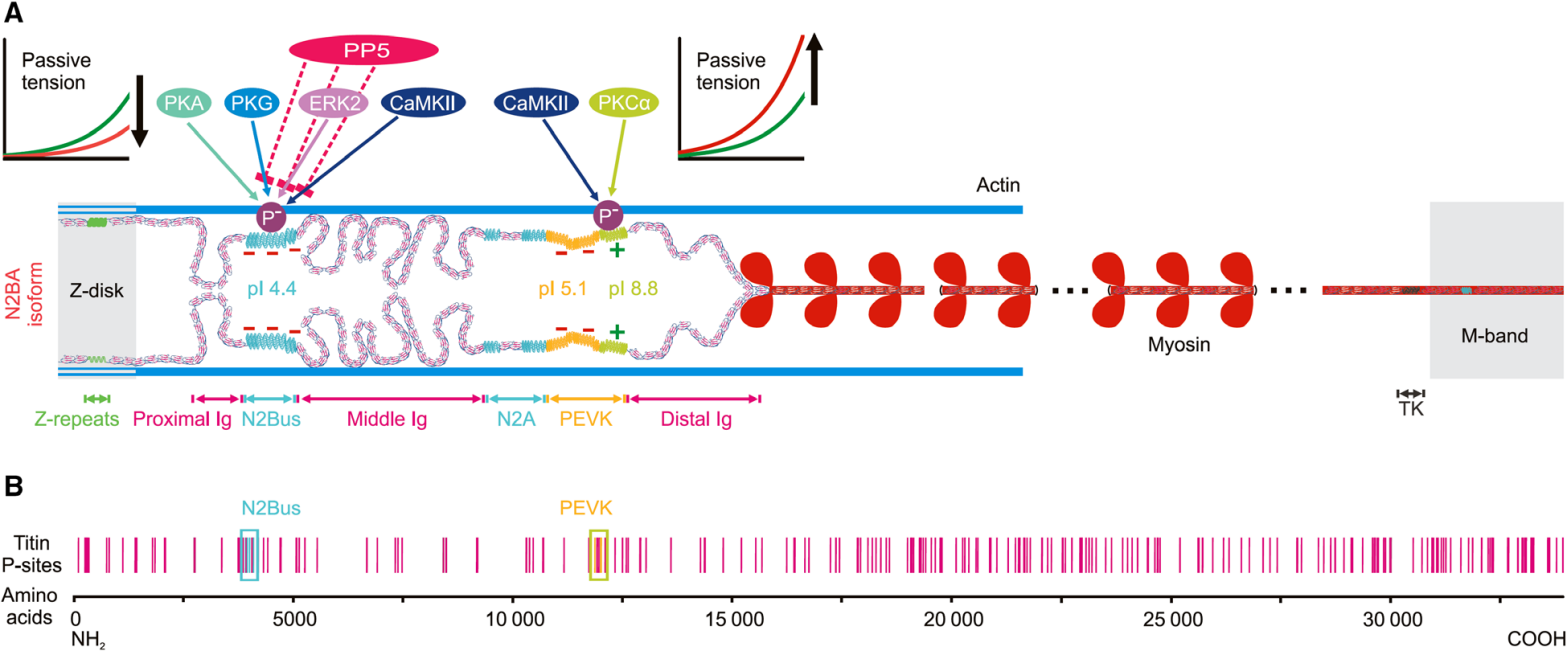
Potential and verified phosphorylation sites in human titin. (A) Layout of the N2BA titin isoform in a cardiac half‐sarcomere, highlighting protein kinases (for details, see main text) and protein phosphatase (PP)5 known to mediate phosphorylation/ dephosphorylation at two distinct molecular spring elements, N2Bus and constitutively expressed PEVK (light green bit of PEVK). Phosphorylation of N2Bus reduces titin‐based passive tension, whereas phosphorylation of PEVK increases it, which is explained by the different net charge of these elements. Constitutive PEVK has a net positive charge (+) and high isoelectric point (pI), N2Bus a net negative charge (−) and low pI. Note that the alternatively spliced PEVK subsegment (yellow bit of PEVK) also has a net negative charge. (B) Known potential phosphosites in human cardiac titin (vertical red bars), from http://www.phosphosite.org 85. Locations of phosphosites verified by site‐specific methods are highlighted (blue and green boxes). TK, titin kinase domain.

Regarding titin, there are five main kinases that have been implicated in the phosphorylation of its molecular spring segment, causing altered titin‐based stiffness (Fig. 3A): protein kinase (PK)A, PKCα, cyclic guanosine monophosphate (cGMP) dependent PKG [gene name: *PRKG1 (*protein kinase cGMP‐dependent 1)], ERK2, and Ca^2+^/calmodulin‐dependent protein kinase IIδ (CaMKIIδ) 6. PKA is activated by cyclic adenosine monophosphate upon β‐adrenergic stimulation. The kinase phosphorylates myofilament and calcium handling proteins in cardiomyocytes to mediate the response of the heart to altered sympathetic activity 71. PKCα (the major PKC isoform in the heart 72) is activated through α_1_‐adrenergic stimulation and has been implicated in many forms of cardiac dysfunction 73, 74, 75. PKG is activated by cGMP following the release of nitric oxide (NO) or natriuretic peptides. Phosphorylation by PKG also regulates cardiomyocyte function in multiple ways, including alterations to myofilament and calcium‐handling protein functions. PKG is importantly involved in pathways of fibrosis and cell survival and it has a protective role in the heart 76. ERK2 is an effector kinase of the Raf1‐MEK1/2‐mitogen‐activated protein kinase pathway and has been implicated in cardiac hypertrophy 77, 78. CaMKIIδ is the predominant CaMKII isoform in the heart regulated by intracellular calcium levels. It has many signaling functions in both the healthy and the diseased heart 79, 80, 81, 82. All of these kinases have the potential to fine‐tune cardiomyocyte passive tension via titin phosphorylation.

Although comparatively little is known about protein phosphatases that dephosphorylate titin, a recent study identified serine/threonine protein phosphatase (PP)5 as an enzyme that binds to the N2Bus region of the I‐band spring 58. PP5 specifically dephosphorylates various sites in N2Bus previously phosphorylated by one of the above‐mentioned protein kinases (Fig. 3A). Transgenic, cardiomyocyte‐specific overexpression of PP5 in mouse heart reduces phosphorylation of N2Bus but not PEVK or Z‐disk titin sites and the mechanical effect is an increase in titin‐based cardiomyocyte passive tension 58. PP5 has less basal activity in cardiomyocytes than PP1 or PP2a, which have many important regulatory functions in these cells 68. However, PP5 is a highly regulated phosphatase that can be activated by various stimuli, for example, by arachidonic acid or HSP90 binding 58. Like PP5, PP1 and PP2a (and alkaline phosphatase) have been used *in vitro* to dephosphorylate cardiac titin segments, including N2Bus and PEVK 83, 84. At present it is unknown whether PP1 and PP2a are involved in the dephosphorylation of cardiac titin *in vivo*, in addition to PP5.

## Which sites in titin are (de)phosphorylated by which enzyme and what is the mechanical effect?

According to phosphoproteomic screens (e.g., http://www.phosphosite.org 85]), many phosphorylation sites are distributed all over the titin molecule (Fig. 3B). More than 300 phosphosites are listed for mouse and human titin 86, representing a huge potential for the regulation of cardiomyocyte structure and function. In light of this large number and the multiple kinases involved in titin phosphorylation, it has been a reasonable approach to measure all‐titin phosphorylation, in order to determine overall trends in the regulation of titin by phosphorylation. For the purpose of this review, we have considered methods involving phosphoprotein staining by sensitive dyes, such as ProQ Diamond, western blotting with anti‐serine/threonine antibodies, and autoradiography after back‐phosphorylation as ‘all titin’ phosphorylation. However, note that none of these methods detects every single phosphosite in a protein. These measurements have provided general insight into the importance of titin phosphorylation in altering titin‐based passive tension, with the obvious disadvantage that they are not able to detect site‐specific phosphorylation events. Considering the overwhelming number of titin phosphosites, the determination of all‐titin phosphorylation arguably has limited informative value.

Only a small number of phosphorylation sites in titin has been verified by site‐specific methods, such as mutagenesis or phosphospecific antibodies. These phosphosites are almost exclusively located within the unique sequences, N2Bus and PEVK (Fig. 3B) 86. As for the PEVK element, only the extreme COOH terminus was included in the previous analyses; this PEVK subsegment is constitutively expressed in all full‐length titin isoforms. One may ask why the site‐specific analysis of titin phosphorylation sites has been limited to these two regions. A reason is that the recombinant expression of the whole titin protein is currently impossible due to its enormous size. In contrast, shorter segments can be readily expressed and analyzed for *in vitro* phosphorylation, and it was a sensible choice to initially focus on N2Bus and PEVK as these regions are established as mechanically active elements in I‐band titin 15, 18, 19. Phosphorylation of these sites was thus considered to have potential to modulate titin‐based passive stiffness. Although this historical bias may skew the importance of phosphorylation at these two segments, differences in phosphorylation at N2Bus and PEVK have been observed between healthy and diseased hearts and have been associated with changes in titin‐based passive tension, supporting the functional relevance of phosphorylation at these two regions. In the future, it will be important to also address the functional implications of phosphorylation sites in titin Ig domain segments (there are many, as indicated in Fig. 3B) and study their possible pathophysiological relevance.

### Confirmed phosphosites in the N2Bus element

**Figure 4:**
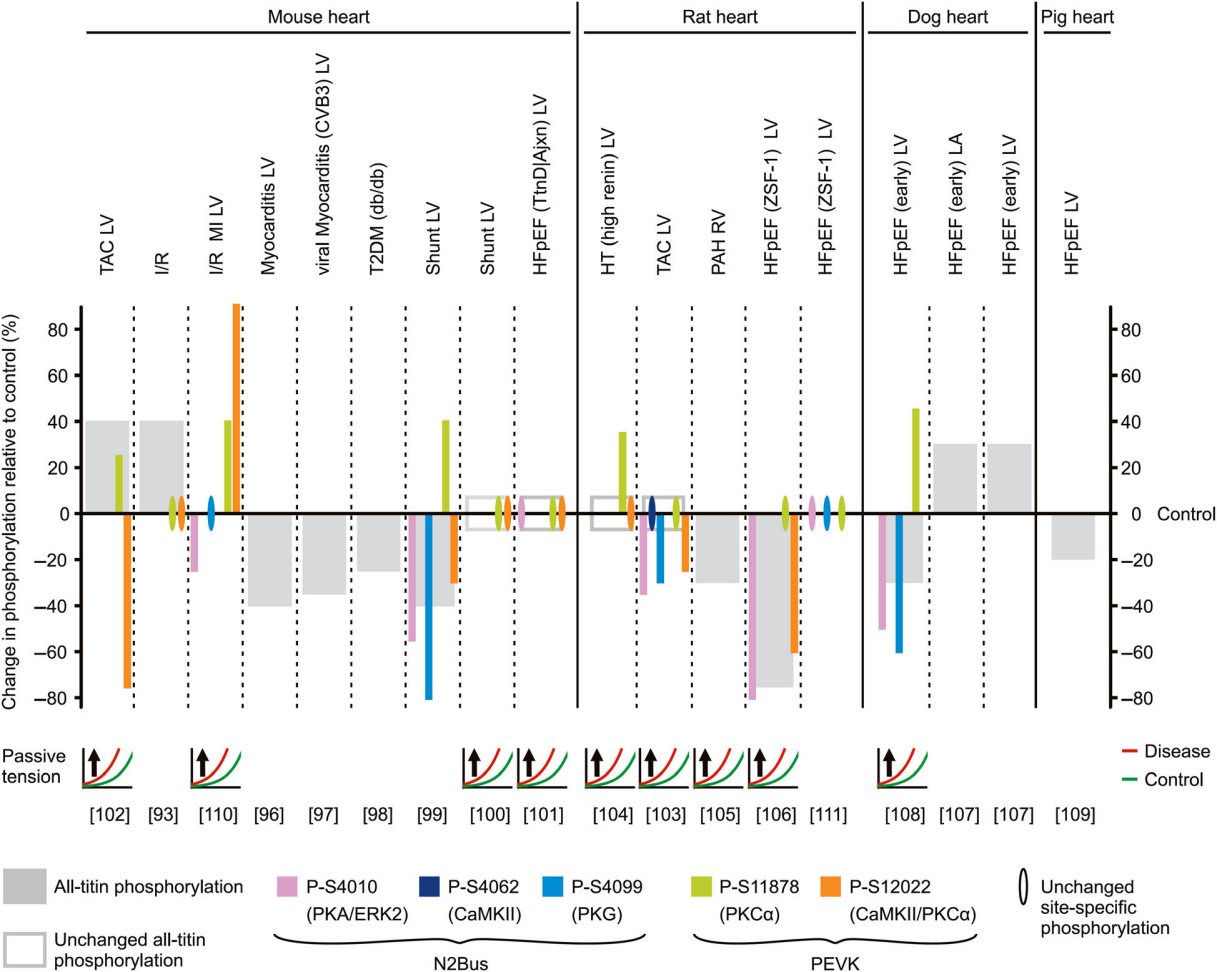
Changes in titin phosphorylation in animal models of heart disease and relationship with alterations in cardiomyocyte passive tension. The size of the bars indicates the relative amount of change in phosphorylation in diseased hearts vs. the respective control, healthy hearts (for some conditions, an average value is shown). The relative changes are as indicated by the respective study authors and therefore, magnitude comparisons between studies may not be plausible. Note the heterogeneity in the direction of change in all‐titin phosphorylation among the different models. Also, note that in failing hearts, the N2Bus element is frequently hypophosphorylated at one or more sites, whereas phosphoserine (S11878) within the PEVK element is usually hyperphosphorylated. Conversely, PEVK site S12022 mostly shows hypophosphorylation. For those studies where titin‐based passive tension was measured, the direction of change (arrow) in disease (red curves) relative to healthy heart samples (green curves) is indicated. Protein kinases known to target individual phosphosites are listed in parentheses. References are in square brackets. TAC, transversal aortic constriction (afterload increase); I/R, ischemia/reperfusion injury; MI, myocardial infarction; T2DM, type 2 diabetes mellitus; Shunt, aorto‐caval shunt model (preload increase); HFpEF, heart failure with preserved ejection fraction; TtnD/Ajxn, deletion of titin segment at I‐band/A‐band junction; HT, hypertension; PAH, pulmonary arterial hypertension; ZSF‐1, Zucker spontaneously hypertensive, fatty‐1 model; LV, left ventricle; RV, right ventricle; LA, left atrium.

**Figure 5:**
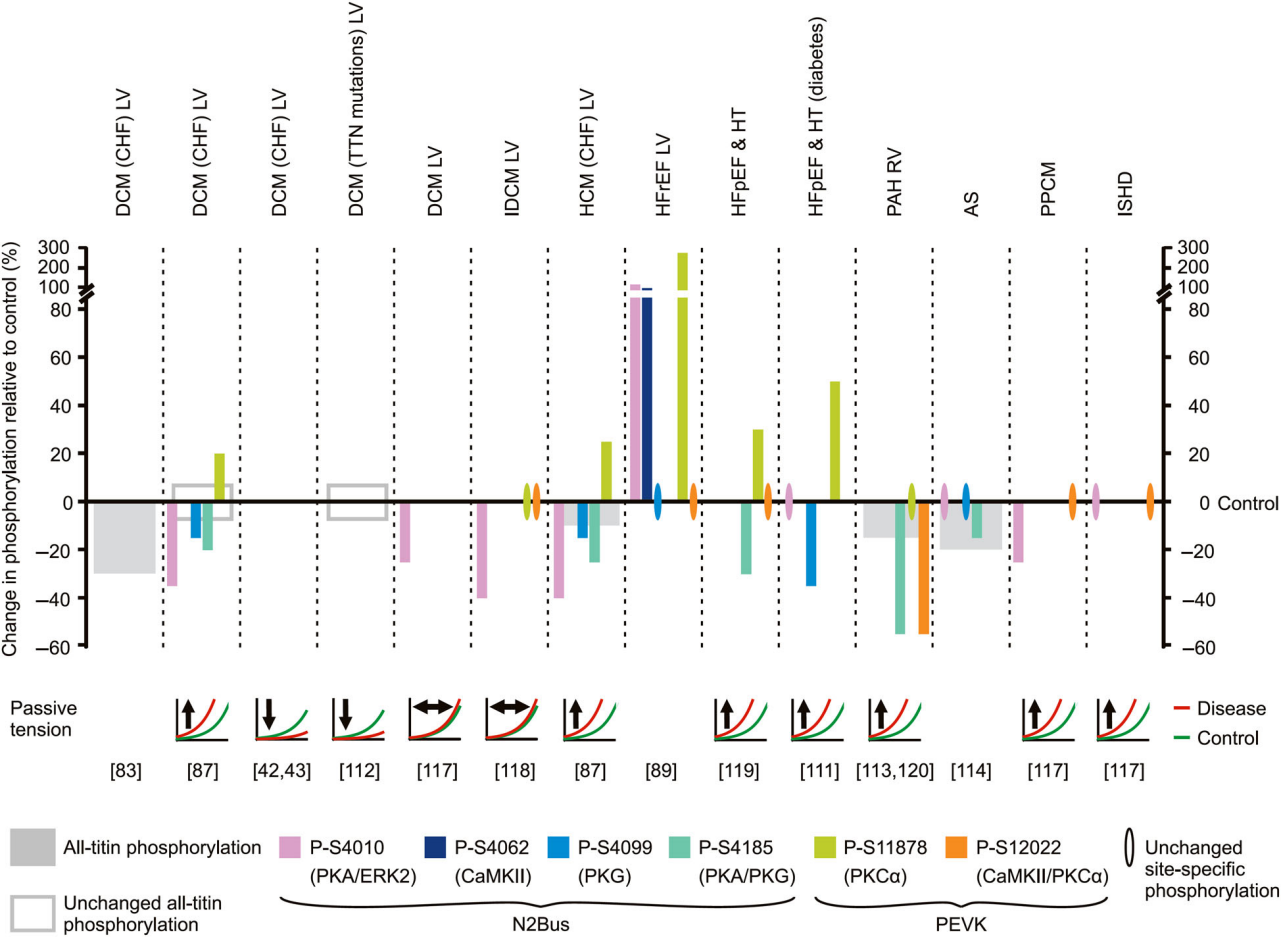
Changes in titin phosphorylation in human failing hearts and relationship with alterations in cardiomyocyte passive tension. The size of the bars indicates the relative amount of change in phosphorylation in failing vs. nonfailing hearts (for some conditions, an average value is shown). The relative changes are as indicated by the respective study authors and therefore, magnitude comparisons between studies may not be plausible. Note the absence of hyperphosphorylation of all‐titin. Also, note the general pattern of hypophosphorylation at one or more N2Bus sites and hyperphosphorylation at PEVK site S11878, whereas phosphorylation at S12022 mostly remains unaltered. For those studies where titin‐based passive tension was measured, the direction of change (arrow) in disease (red lines) relative to healthy heart samples (green lines) is indicated. Protein kinases known to target individual phosphosites are listed in parentheses. References are in square brackets. DCM, dilated cardiomyopathy; CHF, congestive heart failure; IDCM, idiopathic DCM; HCM, hypertrophic cardiomyopathy; HFpEF, heart failure with preserved ejection fraction; HFrEF, heart failure with reduced ejection fraction; HT, hypertensive heart disease; PAH, pulmonary arterial hypertension; AS, aortic stenosis; PPCM, peripartum cardiomyopathy; ISHD, ischemic heart disease. LV, left ventricle; RV, right ventricle.

Within the titin N2Bus segment, several cross‐species–conserved phosphoserines, including S4010, S4062, and S4099, as well as nonconserved (human‐only) S4185 (numbering according to human titin consensus sequence, UniProKB #http://www.uniprot.org/uniprot/Q8WZ42-1), have shown altered regulation in animal models of heart disease and human failing hearts, as will be detailed below (Figs 4 and 5). S4185 was the first phosphosite to be detected in human N2Bus, initially through mutagenesis of recombinant N2Bus protein and back‐phosphorylation/autoradiography 83. A subsequent study confirmed this site via mass spectrometry of back‐phosphorylated, recombinant N2Bus 87. These studies found that S4185 can be phosphorylated by PKA or PKG. Another substrate of PKA is S4010, and PKG also phosphorylates S4099, while several additional (nonconserved) PKA/PKG‐dependent sites are present in N2Bus 87. S4010 is also phosphorylated by ERK2, as are a few other N2Bus sites 88. CaMKIIδ phosphorylates S4062 and presumably additional serines and threonines within N2Bus, as demonstrated by phosphoantibody staining and *in vivo* quantitative phosphoproteomics using mass spectrometry on heart tissues from WT and CaMKII knockout mice, in comparison to hearts from the ‘stable isotope labeling of amino acids’ (SILAC) mouse 89. Dephosphorylation of S4010, S4062, S4099, and S4185 can occur in vivo through PP5 58. The phosphorylation of all these sites has been associated with a decrease in titin‐based cardiomyocyte passive tension 83, 90, the dephosphorylation with an increase 58 (Fig. 3A). The strongest evidence has come from single‐molecule mechanical measurements on recombinant N2B element using AFM force spectroscopy 83, 90. Interestingly, ROS activate both ERK 91 and PP5 92. Thus, under oxidizing conditions, phosphorylation of the N2Bus segment potentially switches to ERK2‐based regulation of phosphosites.

### Confirmed phosphosites in the PEVK element

Regarding the PEVK region, site‐specific verification of phosphosites is currently limited to the constitutively expressed COOH‐terminal portion encoded by *TTN* exons 219‐224 (in the human *TTN* meta‐transcript). Two well‐established phosphoserines are phosphorylated by PKCα, S11878, and S12022 84. At least one of these sites, S12022, is also phosphorylated by CaMKIIδ 89. Both CaMKIIδ and PKCα phosphorylate several other serines/threonines within this PEVK subsegment, as shown by a number of different approaches, including back‐phosphorylation experiments with recombinant PEVK fragments exposed to amino acid exchange, mass spectrometry of in vitro expressed PEVK fragments, and in vivo quantitative mass spectrometry using the SILAC method 84, 89, 93. Interestingly, phosphorylation of the PEVK segment by PKCα increases the passive tension of the cardiomyocytes (Fig. 3A) 84.

### Differences in net charge explain differential changes in stiffness upon phosphorylation

An important difference to the N2Bus element is that the (constitutive) PEVK segment has a different net charge (Fig. 3A). N2Bus has a low isoelectric point (pI = 4.4 in human N2Bus) due to the large number of acidic amino acids, resulting in a net negative charge (as the intracellular milieu of a cardiomyocyte is maintained at a nearly neutral pH of 7.1–7.2 94). The introduction of negatively charged phosphate groups (‘phosphorylation’) could, therefore, increase intramolecular electrostatic repulsion. Thus, the force required to extend the N2Bus segment would be lowered, causing decreased titin‐based passive tension. Conversely, the COOH‐terminal PEVK segment contains many basic amino acids resulting in a high pI of 8.8 (in human titin) and a net positive charge. The introduction of negatively charged phosphate groups would trigger additional intramolecular ionic interactions, causing increased titin‐based passive tension, as the force needed to extend this PEVK subsegment would increase (Fig. 3A). This concept is supported by single‐molecule AFM force‐extension measurements where an increase in the persistence length of the N2Bus element was seen upon phosphorylation with PKG 83, whereas a reduction in the persistence length of the (constitutive) PEVK element appeared upon phosphorylation by PKCα 84. In the case of the PEVK domain, the main effect on stiffness seems to be mediated by phosphorylation of S11878 95. If this concept holds true, one would expect that phosphorylation of the alternatively spliced PEVK region (not yet studied) reduces titin‐based passive tension, as this PEVK subsegment has a pI of 5.1 (in human titin) and a net negative charge (Fig. 3A).

Considering the large size of the I‐band titin spring, phosphorylation of only one site within the N2Bus or PEVK segments may have a negligible effect on overall titin‐based tension. It is more likely that several serines/threonines in N2Bus, PEVK, and/or other titin spring segments must be phosphorylated at one time by a given protein kinase to result in a mechanical effect. An interesting situation arises when a kinase can phosphorylate both N2Bus and PEVK, as is the case for CaMKIIδ 89 (Fig. 3A). In theory, the phosphorylation of both segments together might cause no change in passive tension, as the mechanical effects could cancel out each other. However, mechanical experiments have demonstrated that the passive force of permeabilized cardiomyocytes decreases with ex vivo CaMKIIδ‐treatment 89, 90. Moreover, passive tension was elevated in cardiomyocytes of CaMKIIδ/γ double‐knockout mouse hearts and reduced in those of CaMKIIδ‐overexpressing transgenic mouse hearts, relative to WT mouse cardiomyocytes 89. Thus, the CaMKII‐mediated phosphorylation of N2Bus appears to dominate the mechanical effect. Furthermore, the affinity of CaMKIIδ could be different for the N2Bus and PEVK substrates, or the N2Bus phosphosites may be better accessible than the PEVK phosphosites in a stretched sarcomere. Alternatively, additional unidentified CaMKIIδ‐dependent phosphosites may exist in cardiac titin, which could drive the effect on stiffness.

In summary, site‐specific phosphorylation by several different kinases has been confirmed in the N2Bus and PEVK segments of titin. There is consensus that phosphorylation of the constitutive PEVK segment increases titin stiffness, whereas phosphorylation of N2Bus decreases it. Considering the total number of potential phosphosites in cardiac titin, the verification of other phosphosites (including those in Z‐, A‐ and M‐band titin) by site‐specific methods is needed to provide further insight into the regulation of titin‐based passive tension (and other functions) through phosphorylation. Despite this limitation, phosphorylation of titin spring elements is a well‐established modulator of titin‐based stiffness in cardiomyocytes.

## Phosphorylation of the titin spring in normal and failing hearts

The fine‐tuning of titin‐based passive stiffness through changes in phosphorylation states becomes even more relevant when considering pathological conditions of the heart 6. In heart disease, the regulation of many signaling pathways becomes altered; for example, there is frequent downregulation/deactivation of PKG 76 and upregulation/activation of CaMKIIδ and PKCα 73, 79. Among other things, these changes will alter downstream signaling events, including titin phosphorylation. This could become critical to overall cardiac function, if the changes in titin‐based stiffness become substantial: if myocardial stiffness becomes too high, ventricular filling will be compromised, whereas lower than normal stiffness will blunt length‐dependent activation and thus, impair the Frank–Starling response 8, 29, 30.

Changes in titin phosphorylation have been investigated in animal models of heart disease and in failing hearts of human patients, usually in comparison to healthy/nonfailing hearts. The availability of phosphospecific antibodies against several N2Bus and PEVK sites has been instrumental in these studies. Heart failure is generally defined as a clinical syndrome, characterized by the inability of the heart to deliver enough blood to meet the body's metabolic needs. The HF syndrome thus represents the result of various different cardiac and metabolic disorders. With this in mind, it might be expected that titin phosphorylation varies depending on the metabolic cause of the HF subtype. However, the overall trends of phosphorylation changes in the N2Bus and PEVK segments seem to be relatively consistent across the cardiac diseases studied to date, in both humans and animal models. Importantly, the direction of change can be different for N2Bus and PEVK phosphosites (Figs 4 and 5).

### Which changes in titin phosphorylation occur in which animal models of heart failure?

Numerous animal models (mouse, rat, dog, and pig) have been generated that mimic human heart diseases, for example, ischemia/reperfusion (I/R) injury, myocarditis, hypertension (HT), pulmonary arterial hypertension (PAH), transverse aortic constriction (TAC), type 2 diabetes mellitus (T2DM), chronic cardiac overload (shunt), and heart failure with preserved ejection fraction (HFpEF). These models have provided insight into where (site‐specific) and in what direction (hypo/hyper) the changes in phosphorylation can occur in the diseased states (Fig. 4). These changes have frequently been related to changes in titin‐based passive tension measured in cellular or subcellular cardiac muscle preparations (Figs 4 and 5). If mechanical measurements were performed, the typical result was an increase in passive tension in diseased vs. healthy hearts, with only a few exceptions in human samples. Below we review the changes in titin phosphorylation reported to date, including total titin and site‐specific N2Bus/PEVK phosphorylation, as well as report measured changes in passive tension.

#### Total titin phosphorylation

A majority of HF models presented with reduced cardiac all‐titin phosphorylation compared to healthy controls. In murine studies, two different models of myocarditis 96, 97, a T2DM model 98, and a chronic volume overload (shunt) model 99 all showed reduced all‐titin phosphorylation. However, a similar murine shunt model showed no change in all‐titin phosphorylation 100, as was also the case in a murine model of HFpEF 101. In contrast, all‐titin phosphorylation was significantly increased in left ventricular (LV) samples from mice exposed to TAC 102 or I/R injury 93 (Fig. 4). Unlike in the murine TAC model, no change in all‐titin phosphorylation was seen in a rat TAC model 103 and in the hearts of hypertensive rats 104. Total titin phosphorylation was reduced in right ventricular (RV) samples of a rat model of PAH 105 and was also substantially decreased in a rat model of HFpEF 106, which is different from what was seen in the mouse HFpEF model 101. Dog models of HFpEF also showed conflicting results with regard to all‐titin phosphorylation (Fig. 4). One study showed an increase in all‐titin phosphorylation in left atrial and LV samples from a dog model of HFpEF 107, whereas an earlier study reported a decrease in all‐titin phosphorylation in a different set of LV samples from the same HFpEF dog model 108. A pig model of early HFpEF presenting with hypertension and hyperlipidemia following treatment with deoxycorticosteroneacetate and a western diet showed reduced all‐titin phosphorylation in the hearts, compared to healthy pigs 109.

Apparently contradictory results regarding total titin phosphorylation observed in the animal models might be due to the stage of HF induced in the respective model. For instance, the severity of HF may be different in the mice 102 and the rats 103 exposed to TAC, depending on the level of aortic constriction. Moreover, strain and species differences could underlie the differential changes in titin phosphorylation observed. In any case, the importance of changes in all‐titin phosphorylation is difficult to judge considering the large number of titin phosphosites.

#### N2Bus site‐specific phosphorylation

Site‐specific phosphorylation studies in animals yielded some overall trends but also appeared to be contradictory at times (Fig. 4). The N2Bus segment was found to be either hypophosphorylated or unchanged in all animal species and HF models investigated. PKA/ERK2‐dependent phosphosite S4010 was hypophosphorylated in mice exposed to I/R 110 or chronic volume overload 99, in rat TAC and HFpEF models 103, 106, and in a dog HFpEF model 108. In contrast, no change in S4010 phosphorylation appeared in other mouse and rat HFpEF models 101, 111. PKG‐dependent phosphosite S4099 was also found to be hypophosphorylated in most animal studies 99, 103, 108 except for a mouse I/R model 110 and a rat HFpEF model 111, where S4099 phosphorylation was unchanged. One study on a rat TAC model measured the phosphorylation of CaMKIIδ‐dependent phosphosite S4062, which was unchanged 103. However, as expected, mouse hearts deficient in CaMKIIδ showed hypophosphorylation at this site and transgenic CaMKIIδ‐overexpressing murine hearts had increased phosphorylation at S4062 89. Where measured, an increase in passive tension was seen in all models with N2Bus hypophosphorylation (Fig. 4), consistent with the idea that phosphorylation of the N2Bus segment lowers passive tension of cardiomyocytes. In general, findings on these animal model studies support the concept that decreased phosphorylation of the N2Bus titin segment contributes to the increased myocardial stiffness frequently seen in heart disease.

#### PEVK site‐specific phosphorylation

Site‐specific phosphorylation of the constitutively expressed PEVK titin segment varied greatly between the two phosphosites studied by western blot, and among the animal models used (Fig. 4). All models investigating PKCα‐dependent phosphosite S11878 showed either hyperphosphorylation or no change in the diseased state. Specifically, mice exposed to TAC 102, I/R 110 or chronic volume overload 99, HT rats 104, and a canine model of HFpEF 108 all showed hyperphosphorylation of S11878. In contrast, different studies using murine models of I/R 93 and chronic volume overload 100, a rat TAC model 103, and both a murine and two rat models of HFpEF 101, 106, 111 revealed no change in phosphorylation at S11878.

Differences in phosphorylation of PKCα/CaMKIIδ‐dependent S12022 were substantially more varied, with hyperphosphorylation, hypophosphorylation, or no change in phosphorylation being seen across many different models (Fig. 4). One murine model of I/R showed increased phosphorylation of S12022 110, while another showed no change 93. The difference may potentially be due to differences in the severity of I/R injury in these models. Furthermore, there was no change in S12022 phosphorylation in HT rats 104, murine chronic volume overload 100, and murine HFpEF 101 models, all vs. the respective healthy controls. This is in direct contrast to a similar murine chronic volume overload 99 and a rat HFpEF model 106, where hypophosphorylation at S12022 was observed. Both mouse and rat TAC models also showed hypophosphorylation of S12022 102, 103.

Taken together, constitutively expressed PEVK frequently appears to be hyperphosphorylated at S11878 and hypophosphorylated at S12022 in animal heart disease. Considering the net positive charge of the constitutive PEVK subsegment, hyperphosphorylation of S11878 is consistent with an increase in the passive stiffness of cardiomyocytes, explained by additional ionic interactions within this subsegment of titin upon addition of phosphate groups. Conversely, hypophosphorylation of S12022 would be expected to lower titin‐based passive force. Thus, either altered phosphorylation of S12022 has little effect on titin‐based stiffness (unlike expected) or changes in phosphorylation of S11878—and those at the N2Bus element—may dominate the overall mechanical effect. Additionally, it is likely that altered phosphorylation of titin in HF occurs at PEVK sites not included so far in the analysis using phosphoantibody detection.

In summary, studies on animal models of heart disease suggest an overall decrease in titin phosphorylation compared to healthy control hearts, which may be driven by hypophosphorylation of residues in the N2Bus segment. Concomitantly, there is sometimes hyperphosphorylation of PEVK sites such as S11878 but no change or hypophosphorylation at other PEVK sites such as S12022. The functional net effect of these changes in phosphorylation appears to be an increase in titin‐based passive tension in heart disease.

### Which changes in titin phosphorylation occur in what type of human heart failure?

Human studies into phosphorylation of titin in heart disease are generally in agreement with the animal models in that an overall decrease (vs. nonfailing hearts) occurs in all‐titin phosphorylation and site‐specific N2Bus phosphorylation, whereas hyperphosphorylation occurs at PEVK phosphosite S11878 (Fig. 5). In the majority of cases reported, these changes are also associated with increased cardiomyocyte passive tension.

#### Total titin phosphorylation

Of those studies that measured all‐titin phosphorylation and compared with a control group (mostly nonfailing hearts), four showed a decrease in diseased vs. healthy hearts (Fig. 5). Specifically, all‐titin phosphorylation was decreased 83 or unchanged 87, 112 in the LV of endstage failing human hearts from patients with dilated cardiomyopathy (DCM) compared to nonfailing donor hearts. A moderate decrease in all‐titin phosphorylation vs. control hearts was detected in RV samples from patients with PAH 113 and in LV samples from patients with severe chronic HF due to hypertrophic cardiomyopathy (HCM) 87, as well as in endomyocardial LV biopsies from patients with severe aortic stenosis (AS) 114. Another paper measured the ratio of phosphorylated N2BA to phosphorylated N2B cardiac titin isoforms in myocardial samples from patients with AS and T2DM and found a significant increase relative to nonfailing myocardium, while the N2BA:N2B expression ratio remained unaltered, suggesting hypophosphorylation of the N2B isoform and hyperphosphorylation of the N2BA isoform 115. An earlier paper reported an increased phospho‐N2BA:phospho‐N2B ratio in human HF (including HFpEF) vs. AS myocardium 116. However, this change was accompanied by titin isoform transition toward N2BA of a similar magnitude, suggesting no change in total titin phosphorylation, while there was increased passive tension in HF vs. AS cardiomyocytes. The latter result could be explained, in theory, by differential phosphorylation of the N2Bus and PEVK elements in HF vs. AS, which was not measured in that study. The overall picture emerging from these studies is that human failing hearts frequently have an all‐titin phosphorylation deficit (and never show hyperphosphorylation of total titin), which could drive pathological myocardial passive stiffening.

#### N2Bus site‐specific phosphorylation

A reduction in the phosphorylation of N2Bus sites was usually reported in diseased vs. donor hearts (Fig. 5). Specifically, cardiac titin was hypophosphorylated at S4010 in LV samples from patients with severe DCM 87, 117, idiopathic DCM (IDCM) 118, HCM 87, or peripartum cardiomyopathy (PPCM) 117. Similarly, phosphorylation of N2Bus was decreased vs. controls at phosphosite S4099 in end‐stage failing hearts of patients with DCM 87, HCM 87, or HFpEF associated with T2DM 111. Consistent with these cross‐species–conserved N2Bus sites, S4185 was found to be hypophosphorylated in severely diseased patients with DCM 87, HCM 87, HFpEF associated with HT 119, AS 114, and RV samples from patients with PAH 120. Contradictory, N2Bus was hyperphosphorylated at S4010 and S4062 in a subset of end‐stage failing hearts from transplant patients with HFrEF but signs of hypertrophic cardiomyopathy 89. Whether this result was a consequence of the increased cardiac CaMKIIδ expression and activity reported in these patients remains unclear 89. Aside from this single study, reduced phosphorylation of N2Bus sites appears to be the rule in human HF.

Hypophosphorylation of N2Bus was mostly associated with increased titin‐based passive stiffness in diseased vs. donor hearts (Fig. 5). This observation was made in patients with severe forms of DCM 87, HCM 87, HFpEF associated with HT 119, HFpEF associated with T2DM 111, PAH 113, and PPCM 117. In contrast, some studies found that passive tension was unchanged in LV samples from patients with DCM or IDCM vs. donor hearts 117, 118 or even decreased at the level of the cardiac myofibrils 42, 43, 112. However, information about the titin N2Bus phosphorylation status in these studies was limited or not provided (Fig. 5). Possibly, the switch in titin isoform composition toward N2BA in endstage failing human hearts 42, 43 sometimes reduces titin‐based passive tension despite titin (including N2Bus) hypophosphorylation.

#### PEVK site‐specific phosphorylation

Hyperphosphorylation of PEVK phosphosite S11878 was seen in some human disease studies (Fig. 5). Specifically, phosphorylation of S11878 was increased in LV samples from patients with severe DCM 87, 89, HCM 87, and HFpEF associated with HT 119 or T2DM 111 vs. the respective nonfailing controls. These changes were accompanied by an increase in titin‐based passive tension. In a few studies on IDCM (LV) and PAH (RV), S11878 phosphorylation was unaltered 118, 120. Interestingly, in most reported cases, the phosphorylation status of PEVK phosphosite S12022 was unaltered in failing vs. donor hearts (Fig. 5). In RV samples of PAH patients, S12022 was even hypophosphorylated and despite unaltered S11878 phosphorylation, cardiomyocyte passive tension was increased 120. Again, the hypophosphorylation of N2Bus sites may be the driving factor that raises titin‐based stiffness in the hearts of these patients.

In summary, titin is consistently hypophosphorylated at one or more N2Bus sites in failing human vs. nonfailing hearts. Frequently, titin is also hyperphosphorylated at PEVK residue S11878, but not at S12022. Such relatively consistent states of phosphorylation from different heart disease stages may be somewhat unexpected; however, the direction of change is consistent, at least in part, with previously shown alterations in the expression/activity of protein kinases and phosphatases in heart disease, especially PKCα and PKG 73, 76 as well as PP5 58. Interestingly, these findings are somewhat similar to what has been observed during acute exercise 121. One may speculate that this similarity is also an indication of the additional work done by the heart in a diseased state. In any case, these differential alterations in titin phosphorylation are predicted to cooperatively increase titin‐based passive stiffness and thus, myocardial stiffness in disease.

## Potential for human therapy

Post‐translational modifications of titin that alter titin‐based myocardial passive stiffness represent a potential target for therapeutic intervention in HF patients with overly stiff hearts, such as HFpEF. Strong evidence has been obtained for the beneficial effect of enzyme‐mediated alterations in titin‐based passive tension in mechanical measurements on isolated myocardial samples. When skinned cardiomyocytes isolated from human hearts are treated ex vivo with PKA 59, 122, PKG 83, 116, 123, or CaMKIIδ 120, their passive tension drops. The PKA and PKG effects appear to be particularly strong in cardiomyocytes from failing hearts where titin is hypophosphorylated at the N2Bus element, presumably due to reduced expression/activity of these enzymes in HF (for a recent review, see 86). Attempts have been made to normalize myocardial (titin‐based) passive tension, and thereby improve diastolic filling, by boosting the cGMP‐PKG axis *in vivo*. Studies on HF patient‐mimicking, large animal models are ongoing 109, 124, 125. Work on a dog model of early HFpEF demonstrated acute beneficial effects on cardiomyocyte stiffness and LV diastolic distensibility upon intravenous administration of sildenafil, an inhibitor of phosphodiesterase 5A (PDE5A; a cGMP‐degrading enzyme), and brain natriuretic peptide (an activator of particulate guanylate cyclase), concomitant with a rise in total titin phosphorylation, which were explained by increased cGMP expression 124. However, PDE5A does not appear to be expressed at appreciable levels in cardiac tissue lysates from either normal or failing hearts 126, suggesting it may not be a good therapeutic target in human HF. Whether another cGMP‐selective phosphodiesterase, PDE9A, is more highly expressed in failing human cardiomyocytes, is a controversial issue requiring further study 127, 128, as do the potential benefits of inhibiting PDE9A on titin phosphorylation and LV passive stiffness in human HF. Furthermore, recent work demonstrated no benefit on LV compliance from boosting cGMP‐PKG signaling by intravenous administration of BAY 41‐8543, a pharmacological stimulator of soluble guanylate cyclase, in a porcine model of early HFpEF 129. In this study, total titin phosphorylation and site‐specific phosphorylation of both N2Bus and PEVK sites were increased compared to untreated pigs, suggesting multiple unexpected effects on titin phosphorylation upon stimulation of the cGMP‐PKG pathway. These results dampen the enthusiasm for targeting the cGMP‐PKG axis in attempts to normalize myocardial passive stiffness via effects on titin phosphorylation. Whether other avenues of cGMP‐PKG signaling may be more promising as potential targets for therapy in this context remains to be seen. Moreover, signaling pathways of other titin‐targeting protein kinases and phosphatases still need to be explored for possible effects on titin phosphorylation through pharmacological manipulation.

## Conclusions and outlook

The rapid regulation of titin stiffness helps to dynamically couple cardiac myofilament activity to the prevailing hemodynamic demands of the circulation under physiological and pathophysiological conditions. The main focus of this review has been the regulation of cardiac titin stiffness by two types of PTMs, oxidation and phosphorylation. As discussed, the last decade has seen much progress in our understanding of how oxidation and phosphorylation of titin spring elements alter titin stiffness, and in which direction. Mechanistic insight has come from *in vitro* studies on single isolated cardiomyocytes, isolated titin molecules, or recombinant titin fragments. Phosphospecific antibodies have allowed for the evaluation of titin phosphorylation states at individual amino acids in diseased vs. healthy hearts, including human failing hearts, and the correlation with changes in titin‐based spring force. Information on phosphorylation changes *in vivo* is available for the two titin spring elements N2Bus and (constitutive) PEVK, but is lacking for the Ig domain segments, which do contain many (potential) phosphosites. Moreover, ‘classical’ tests to probe the functional relevance of a PTM within a molecule of interest have not been performed for titin, for example, the knock‐in of the protein in a transgenic mouse model with the phosphosite in question being mutated to a constitutively phosphorylated/phosphomimicking or nonphosphorylatable residue and the phenotypic changes being quantified. In fact, it is questionable whether such an approach would be sensible for a molecule the size of titin, especially regarding the effect on titin stiffness, because a mechanical effect presumably requires the biochemical modification of several titin residues at once. Conversely, it has been useful to quantify site‐specific titin phosphorylation in mouse models where a relevant protein kinase (CaMKII) was ablated or overexpressed 89 or a relevant phosphatase (PP5) overexpressed 58. However, these studies cannot address the dynamic changes in titin phosphorylation likely occurring swiftly under physiological and pathological conditions.

We still lack crucial knowledge on several issues, including what is the relative importance of titin isoform switching vs. PTMs for the regulation of titin stiffness *in vivo* and whether the changes in titin phosphorylation observed in failing hearts can be the primary cause of heart disease or instead represent compensatory adaptations. Oxidative changes in titin have thus far been examined almost exclusively *in vitro*, which calls for new studies to establish the degree and exact location of titin oxidation *in vivo*. Furthermore, the relevance of titin oxidation in heart disease is largely unknown. Other PTMs (such as arginylation 130) may also alter titin‐based passive stiffness and warrant further investigation into the role they may play in both healthy and diseased hearts. Finally, PTMs of titin that alter titin‐based myocardial passive stiffness represent a promising potential target for therapeutic intervention in HF patients with overly stiff hearts (such as in HFpEF), despite recent setbacks in some preclinical studies regarding to the role of cGMP‐PKG activation for myocardial stiffness *in vivo*. However, new approaches have already shown that titin phosphorylation and titin compliance can be increased in animal models by targeting various other intracellular pathways 111, 131, 132, 133. Results of these studies suggest that the correction of mechanically relevant PTMs of titin in failing hearts can reduce pathological wall stiffness and normalize diastolic function. The further identification of these PTMs and their impact on titin‐based passive tension will be instrumental in substantiating these achievements and translating the findings to humans.

## Conflicts of interest

The authors declare no conflict of interest.

## Author contributions

All authors contributed toward literature review, manuscript generation, critical revision of manuscript, and illustration. Illustration generation was done by FK.
